# Electrospun PEDOT-Based Meshes for Skin Regeneration

**DOI:** 10.3390/polym17162227

**Published:** 2025-08-15

**Authors:** Alexandra I. F. Alves, Nuno M. Alves, Juliana R. Dias

**Affiliations:** CDRSP-IPLeiria—Centre for Rapid and Sustainable Product Development, Polytechnic Institute of Leiria, 2430-028 Marinha Grande, Portugalnuno.alves@ipleiria.pt (N.M.A.)

**Keywords:** in situ chemical polymerization, PEDOT, wound dressing, conductive electrospun fibers, skin regeneration

## Abstract

The application of conductive polymers in wound dressings presents great potential for accelerated wound healing since their high electrical conductivity and biocompatibility facilitate the delivery of external electrical stimuli to cells and tissues, promoting cell differentiation and proliferation. Electrospinning is a very straightforward method for the preparation of polymeric wound dressings capable of mimicking the extracellular matrix of skin, promoting hemostasis, absorbing wound exudate, allowing atmospheric oxygen permeation and maintaining an appropriately moist environment. In this work, in situ chemically polymerized poly(3,4-ethylenedioxythiophene) (PEDOT) was achieved through hyaluronic acid-doping. The synthesized PEDOT was used for the production of conductive and biodegradable chitosan (CS)/gelatin (GEL)/PEDOT electrospun meshes. Additionally, the randomly aligned meshes were crosslinked with a 1,4-butanediol diglycidyl ether and their physicochemical and mechanical properties were investigated. The results show that the incorporation of a conductive polymer led to an increase in conductivity of the solution, density and fiber diameter that influenced porosity, water uptake, and dissolvability and biodegradability of the meshes, while maintaining appropriate water vapor permeation values. Due to their intrinsic similarity to the extracellular matrix and cell-binding sequences, CS/GEL/PEDOT electrospun nanofibrous meshes show potential as conductive nanofibrous structures for electrostimulated wound dressings in skin tissue engineering applications.

## 1. Introduction

The skin represents the first line of defense of the human body against the external environment, protecting it against pathogens and physicochemical and thermal aggressions [1,2,3]. Skin wound types are greatly aggravated by increasing depth of the cutaneous injury; while partial-thickness injuries often do not require grafting and can self-heal if properly debrided and treated with antimicrobial dressings. In full-thickness injuries, the skin’s self-regenerating potential is greatly reduced and generally requires replacement of the epidermal barrier by transplantation of skin grafts, keratinocyte suspensions/sheets or dermal-epidermal skin substitutes [3,4,5]. In healthy human skin, the epidermis maintains a transepithelial potential (TEP) ranging between 10 and 60 mV [6]. An injury to the skin’s epidermis originates a TEP short circuit that is measurable by a direct current efflux from the wound between 1–10 µA/cm^2^, which corresponds to a relatively steady local electric field of 40–200 mV/mm [6]. This electric field persists until wound re-epithelization is concluded, serving as guidance for cell migration. Electrical stimulation for wound healing allows the delivery of a low-intensity electric current, working effectively in the enhancement of fibroblast and keratinocyte migration and proliferation, upregulating fibroblast growth factor and cytokine secretion and inducing fibroblast to myofibroblast transdifferentiation [6,7]. In the last two decades, conductive polymers (CPs) such as PEDOT, polyaniline (PANI) and polypyrrole (PPy) have received great interest in the biomedical, biosensors and electronics fields due to their high stability, tunable chemical and electrochemical properties, biocompatibility and porosity [8,9,10,11,12]. Particularly for tissue engineering applications, CPs are an emergent class of electroresponsive biomaterials that allow direct control and delivery/triggering of electrical, electrochemical and electromechanical stimuli to cells/tissues [9,13,14,15].

The integration of intrinsically CPs in wound dressings presents an innovative opportunity for the enhancement of wound healing by delivering physical or biochemical cues to the wound site, through the use of an external electrical stimulus. While passive dressings primarily offer mechanical protection and moisture retention, PEDOT-based materials bring additional electroactive functionality. Specifically, they can deliver gentle electrical cues that mimic endogenous bioelectric signals in skin, thereby influencing key cellular behaviors, such as fibroblast migration, keratinocyte proliferation and collagen synthesis, and ultimately accelerating wound healing and reducing scar formation [7,8].

CPs have been proven to deliver these cues in a localized, controlled and efficient manner, permitting control over the microenvironment [15]. For electrical stimulation in tissue engineering, conductive scaffolds must ideally be biodegradable, bioresorbable and sterilizable and possess adequate dimensions, low resistance and high stability [8,13]. However, despite being biocompatible, CPs are not intrinsically biodegradable nor bioresorbable, presenting quite limited processability due to their brittleness once synthesized [13,16]. To overcome these processing limitations and biodegradability issues, CPs are frequently combined with biomolecules or biocompatible polymers via doping, formation of composites, co-polymerization or blending. The combination of CPs and other biopolymers during the polymerization process is a very straightforward method that allows countless possibilities in the combination of materials’ qualities for a precise tailoring of CP-based composites’ biocompatibility and physical properties [11,13,17,18]. CPs’ ability to be electrically switched between an oxidized and a reduced state, during synthesis, also allows the loading/unloading of charged molecules to/from the polymer’s matrix, functioning both as a stimulable drug delivery system and a bioactive scaffold [15]. When CPs are synthesized, either chemically or electrochemically by oxidation of the monomer, there is concomitant incorporation of a negatively charged dopant molecule; thus, by selecting bioactive molecules as their negatively charged dopants, the polymers can be specifically functionalized through the incorporation of, for example, ECM components [19,20]. Despite the functionalization of CPs by doping of biological dopants or incorporation of biological molecules (e.g., growth factors or ECM components), the preparation of clinically relevant CP-based tissue scaffolds is still challenging and very dependent on mechanical and topographical features, which can be greatly affected by the synthesis conditions and dopants used [21].

Physical guidance cues, such as the topographical characteristics, can also be used to make a CP better suited for wound dressing applications. However, translating these materials from bench to bedside requires carefully addressing several key challenges, namely, ensuring sterilization compatibility (low-temperature sterilization techniques can preserve the electroactive properties), evaluating biocompatibility and degradation products (long-term studies) and regulatory approval (adherence to relevant regulatory frameworks and standards). Electrospinning is an inexpensive biofabrication technique that permits the engineering of nanofibrous scaffolds with specific requests in terms of porosity and surface morphology, mimicking the ECM architecture and directly influencing cell adhesion and proliferation, protein expression and matrix remodeling [2,22]. Wound dressings obtained through electrospinning have improved properties when compared to conventional dressings, namely, promoting hemostasis, wound exudate absorbability, semi-permeability, conformability to the wound contour, functional ability and scar-free wound healing [1,23]. An adequate porosity and pore size, which are easily tailorable properties, allow nutrient exchange and the removal of waste products and can provide physical protection against microorganisms due to their small pore size.

In this work, a widely studied polythiophene derivative, PEDOT, was blended with a chitosan/gelatin (CS/GEL) solution to produce randomly aligned conductive nanofibrous meshes with adequate characteristics for wound dressing applications. CS and GEL are natural biodegradable polymers that evidence great results as substrates for wound dressing applications due to their biocompatible, hemostatic, antimicrobial and antibacterial properties [1,2].

PEDOT was synthesized through in situ chemical polymerization of EDOT in acid solution in the presence of a polycationic biopolymer—chitosan-, a biological dopant—hyaluronic acid-and a counterion, polystyrene sulfonic acid sodium salt (PSS), with the oxidant iron (III) chloride hexahydrate (FeCl_3_.6H_2_O). The oxidant does not only act as an electron scavenger for polymerization and doping but also as the source for the counterion tetrachloroferrate-III (FeCl_4_^−^ from FeCl_3_.6H_2_O). CS is a deacetylated compound of chitin, capable of binding itself to the polymer chain during the doping process, improving the final composite’s biocompatibility, processability and surface roughness [13,24]. Hyaluronic acid (HA) is a long and hydrated glycosaminoglycan found in almost all extracellular tissues spaces in the body, capable of binding to the polymer chain, due to its negative charge, serving as a biological dopant [21]. Although it displays angiogenic potential, HA is responsible for the decrease in conductivity of conductive polymer [13,19,20]. PSS is a key component in acting as the counterion (PSS^−^) for PEDOT, permitting its dispersion in water. Despite sometimes being called a dopant in the literature, it cannot be the dopant as it does not have an oxidative effect [25].

The resulting composite is to be later incorporated in a CS/GEL blend solution for electrospinning of nonwoven nanofibers, rendering them conductive and capable of delivering stimuli directly to cells. Chitosan has several active functional groups, such as hydroxyl and amino groups, acting as a cationic polyelectrolyte in acidic medium in the pH range 2–6 due to the presence of amino groups [26]. Gelatin derives from the partial hydrolysis of collagen and contains the arginine–glycine–aspartic acid (RGD) sequence, which is essential for stable connections between cells and the surrounding ECM, enhancing cell adhesion through interactions with integrin [27]. However, it is a water-soluble protein, thus requiring crosslinking. Epoxy-based crosslinkers, such as 1,4-butanediol diglycidyl ether (BDDGE), appear as a less toxic alternative for the commonly used aldehydes for biomedical application, reportedly also being used as a crosslinker for hyaluronic acid dermal fillers [28,29,30].

This study analyzes the viability of producing electrospun conductive CS/GEL nanofibers through the incorporation of in situ chemically polymerized PEDOT, evaluating the benefits of polymerization in the presence of CS and HA as a means to overcome brittleness and biodegradability and in the presence of PSS as a means to overcome dispersion issues through assessment of the physicochemical properties of the produced meshes as scaffolds for skin tissue engineering applications.

## 2. Materials and Methods

### 2.1. Materials

Gelatin powder from porcine skin (type A, 300 bloom, 60 mesh, pharmaceutical grade) was generously offered by Italgelatine (Santa vittoria d’Alba, Italy). Chitosan (deacetylation degree: 85%) was purchased from Acros Organics (M_w_: 100,000–300,000) (Geel, Belgium), glacial acetic acid (AA) was purchased from PanReac AppliChem (Barcelona, Spain), 3,4-ethylenedioxythiophene (EDOT, purity: 97%) and triethylamine (TEA, purity: ≥99.5%) were purchased from Sigma Aldrich (St. Louis, MO, USA). For the polymerization process, hyaluronic acid sodium salt from *Streptococcus equi* (purity: >99%) was purchased from Sigma-Aldrich (St. Louis, MO, USA), poly (sodium-p-styrene sulfonate) was purchased from Acros Organics (M_w_: 70.000) (Belgium) and iron (III) chloride hexahydrate (purity: 97%) was purchased from from Sigma-Aldrich (St. Louis, MO, USA). The crosslinker, 1,4-butanediol diglycidyl ether (BDDGE, purity: 96%), was purchased from Alpha Aesar (Haverhill, MA, USA) and lysozyme from chicken egg white (protein ≥ 90%, ≥40,000 units/mg protein) was purchased from Sigma-Aldrich (St. Louis, MO, USA). All materials used were of reagent grade and without further purification.

### 2.2. Chemical Synthesis of Poly(3,4-Ethylenedioxythiophene) (PEDOT)

For the chemical polymerization of EDOT, two solutions were prepared. The first solution was prepared by dissolving chitosan (3.6 wt.%), hyaluronic acid (3 wt.%) and polystyrene sulfonate (3 wt.%) in AA 50 *v*/*v*.%, succeeded by the addition of EDOT (3.6 wt.%) and the second solution by dissolving FeCl_3_.6H_2_O in double-distilled water (ddH_2_O) for an oxidant/monomer ratio of 9:1. The oxidant solution was added dropwise to the monomer solution and left at room temperature, with stirring, for 96 h. After that time, the acid solution was neutralized with sodium hydroxide (NaOH) 3 M, allowing the precipitation of the CS/PEDOT composite, followed by centrifugation and 10 washing cycles with ddH_2_O to achieve the cleanliness of the solution’s supernatant. The resulting composite was dried for 24 h at 37 °C.

### 2.3. Electrospinning of Crosslinked Chitosan/Gelatin/PEDOT and Control Nanofiber Meshes

Electrospun meshes were produced from two solutions: a CS/GEL solution prepared by dissolving chitosan (3.6 wt.%) and gelatin (14.4 wt.%) in AA 70 *v*/*v*.%, and a CS/GEL/PEDOT solution prepared by dissolving chitosan (3.6 wt.%) and gelatin (14.4 wt.%) in AA 70 *v*/*v*.% followed by the re-dispersion of the CS/PEDOT composite (3.6 wt.%). To both solutions, 2 wt.% of TEA was added, donating electrons to the carboxylic group of the gelatin molecule, which allows the reaction between them and the epoxide groups of BDDGE [1]. PEDOT dispersion was carried out by probe-type sonication (UP200Ht Ultrasonic Processor, Hielscher, Teltow, Germany) for 16 h in a small water bath and stirring at 30 °C. After that time, BDDGE 4 wt.% was added to both solutions promptly before electrospinning, as a means to avoid the loss of configuration that is usually induced by a crosslinking bath. The resultant samples were left at 37 °C for 24 h, 48 h and 72 h to evaluate the crosslinking process.

Samples of CS/GEL/PEDOT and CS/GEL electrospun meshes were produced using a home-made electrospinning apparatus, consisting of a high voltage power supply (73030, Genvolt, Shropshire, UK), a syringe pump (Legato^®^ 101, Kd Scientific, Holliston, MA, USA) and a grounded copper collector.

Due to the distinct conductive nature of both solutions, the parameters used were a constant flow rate of 0.3 mL/h, a needle-tip to collector distance of 12 cm and 20 kV and 30 kV of voltage for the CS/GEL and CS/GEL/PEDOT, respectively, and sample collection lasted for 1 h. The PEDOT-containing solution would not form nanofibers at tensions below 30 kV, as an accumulation of solution would appear on the tip of the needle. However, for the CS/GEL control solution, the voltage could not be 30 kV due to the lower conducting nature of this solution, as the maximum threshold for nanofiber production was 20 kV, in order to prevent electrospraying. Both samples of electrospun meshes were obtained at room temperature (RT) and relative humidity in the range of 40–50%.

### 2.4. Physicochemical Characterization

#### 2.4.1. Apparent Density and Mesh Porosity

The apparent mesh density (ρ_apparent_) and mesh porosity of the CS/GE/PEDOT and control electrospun meshes were calculated using Equations (1) and (2), respectively [31]. The samples were cut with similar, yet slightly variable values of height and width, measured with a regular ruler, and the meshes’ thickness was measured using a micrometer at five different positions in order to obtain the average measurement.

The bulk density parameter (ρbulk composite) was calculated using Equation (3) [32], in which wgelatin, wchitosan and wPEDOT correspond to the mass fractions of gelatin (14.4%), chitosan (3.6%) and PEDOT (3.6%), respectively. The bulk densities of gelatin (ρgelatin), chitosan (ρchitosan) and PEDOT (ρPEDOT) used were 0.72, 1.15 and 1.331, as provided by the suppliers. Five samples for each condition were analyzed.

(1) $$\rho \mathrm{apparent}\;{(g.cm}^{-3})\;\frac{\mathrm{mesh}\;\mathrm{mass}\;(g)}{\mathrm{mesh}\;\mathrm{thickness}\;(\mathrm{cm}).\;\mathrm{mesh}\;\mathrm{area}\;({\mathrm{cm}}^{2})}$$

(2) $$\mathrm{Mesh}\;\mathrm{porosity}\;(\%)=\left(1\;-\;\frac{\mathrm{Apparent}\;\mathrm{mesh}\;\mathrm{density}\;\left(g.{\mathrm{cm}}^{-3}\right)}{\rho \mathrm{bulk}\;\left(g.{\mathrm{cm}}^{-3}\right)}\right)\cdot 100\%$$

(3) $$\frac{1}{\rho \mathrm{bulk}\;\mathrm{composite}\;\left(g.{\mathrm{cm}}^{-3}\right)}=\frac{\mathrm{wgelatin}\;}{\rho \mathrm{gelatin}\;\left(g.{\mathrm{cm}}^{-3}\right)}+\frac{\mathrm{wchitosan}}{\rho \mathrm{chitosan}\;\left(g.{\mathrm{cm}}^{-3}\right)}+\frac{\mathrm{wPEDOT}}{\rho \mathrm{PEDOT}\;\left(g.{\mathrm{cm}}^{-3}\right)}$$

#### 2.4.2. Morphology and Fiber Diameter

The morphology of each electrospun fibrous mesh condition was examined by scanning electron microscopy (SEM) (VEGA3-LM, Tescan, Brno, Czech Republic). Before the examination, samples were fixed on SEM stubs and sputter-coated with a gold/palladium (Au/Pd) film, using a sputter coater (Quorum Technologies, East Sussex, UK). SEM images were used to evaluate the fiber diameter distribution by analyzing three individual samples and randomly measuring 50 fibers of each condition using ImageJ^®^ software (version 1.54 g).

#### 2.4.3. Mesh Structure

To evaluate the chemical composition of the CS/GEL and CS/GEL/PEDOT electrospun meshes and eventual structural changes derived from chemical reactions, Fourier transform infrared (FTIR) spectroscopy with attenuated total reflectance (ATR) was used. FTIR-ATR analyses were carried out using an FTIR-ATR spectrometer (Alpha-P Brucker, Ettlingen, Germany), in the range of 4000–500 cm^−1^, at a 4 cm^−1^ resolution with 64 scans.

#### 2.4.4. Electrical Conductivity and pH

Electrical conductivity of electrospinning solutions was measured before electrospinning at 25 °C using a multiparametric meter (901, ScanSci, Porto, Portugal) with a K10 probe, and the results were reported as average conductivity (*n* = 5). The electrospun meshes sheet resistance at RT was measured (*n* = 5) through Four-Point Probe Plus system (T2001A5, Ossila, Leiden, The Netherland). The pH of the same solutions was measured using a P11/HA probe, and the results were reported as average pH (*n* = 5).

#### 2.4.5. Water Vapor Permeability

To evaluate the water vapor permeation rate of the electrospun meshes, a modified ASTM method as described by the ASTM E96/E96M-16 water method was used [33]. Briefly, glass bottles were filled with 5 mL of distilled water and sealed with the electrospun meshes, covering their openings entirely. The useful area for water vapor permeation was 0.866 cm^2^. To quantify the evaporation of water through the openings, each set was weighed and kept at 32 °C for 24 h; after this time their weight was measured again and recorded to quantify the amount of water evaporated. Five samples for each condition were analyzed.

#### 2.4.6. Dissolvability and Water Uptake

To assess the water uptake and dissolvability of the control and conductive meshes, the samples were dried for 24 h before weight measurement to ensure absolute dryness, followed by incubation in distilled water. After 24 h of incubation, the samples were removed from the distilled water solution, excess water was removed and the samples were weighed again to evaluate the water uptake (Equation (4)). Then, the samples were dried for an additional 24 h period at 37 °C and weighed to evaluate their dissolvability (Equation (5)). In order to understand the influence of the crosslinking extent in mesh dissolvability, the samples were left to crosslink at 37 °C for 24 h, 48 h and 72 h. At least fifteen samples of each condition were analyzed.

(4) $$\mathrm{Water}\;\mathrm{Uptake}\;(\%)=\frac{W_{w}-W_{d}}{W_{d}}\cdot 100$$

where W_w_ is the wet weight and W_d_ is the dry weight.

(5) $$\mathrm{Dissolvability}\;(\%)=\frac{W_{0}-W_{d}}{W_{0}}\cdot 100$$

where W_0_ is the initial weight and W_d_ is the dry weight.

#### 2.4.7. Hydrolytic and Enzymatic Degradation

The hydrolytic and enzymatic degradation of both the control (CS/GEL) and conductive (CS/GEL/PEDOT) samples (5 × 10 mm) were evaluated for 28 days. For the hydrolytic degradation group, both samples were immersed in 5 mL of PBS/0.02 *wt*/*v*.% sodium azide (as bacteriostatic agent), while for the enzymatic degradation group, samples were immersed in the same PBS solution, to which lysozyme from chicken egg white was added. Lysozyme is responsible for chitosan and gelatin degradation and it exists in human body fluids and tissues at various concentrations, ranging from 4 to 13 mg·L^−1^ in serum and from 450 to 1230 mg·L^−1^ in tears [34,35]. Based on the concentration of lysozyme in human serum, a concentration of 7 mg·L^−1^ was used. Samples were incubated in an orbital shaker incubator KS 4000i control (IKA, Staufen, Germany) at 37 °C, 100 rpm for 28 days. Hydrolytic degradation samples’ media were changed weekly, while enzymatic degradation samples’ media were changed twice a week, to ensure enzyme activity. During the experiment, five time-points were determined at 3, 7, 14, 21 and 28 days, at which the samples (*n* = 5) from both batches were collected, and their wet weight was measured and recorded. After that, they were placed to dry for 24 h at 37 °C. At the end of that time, their dry weight was measured to calculate the mesh degradation.

#### 2.4.8. Contact Angle

To assess the hydrophilicity of the electrospun samples, the static contact angle was measured using an optical tensiometer (Attension Theta Lite TL100, Espoo, Sweden). The water contact angle was measured through the recording of the spread of one droplet on the surface of each sample for 1 min and calculating the contact angle with the OneAttension^®^ Software (version 2.1 (r3963)). At least five samples of each condition were analyzed.

#### 2.4.9. Mechanical Properties

The tensile strength at break, the elongation at break and Young’s modulus of control and conductive samples were determined in the wet state using a texturometer (TA.XT PlusC, Stable Micro System, Surrey, UK) with a 50 N load cell. Mechanical tests were carried out at room temperature using a gauge length of 5 mm and a test speed of 1 mm.s^−1^. At least fifteen individual samples from each group were tested.

### 2.5. Statistical Analysis

All data points were expressed as mean ± standard deviation (SD). Statistical analysis (Kolmogorov–Smirnov, Levene’s and *t*-test) was carried out using IBM SPSS Statistics 20.0 with a 95% confidence level. The results were considered statistically significant when *p* ≤ 0.05 (*).

## 3. Results and Discussion

### 3.1. Morphological and Structural Characterization

Matrix-like scaffolds provide topographical cues for guided cell proliferation while providing mechanical support for tissue regeneration. A scaffold should have high porosity (>90%), a large surface area-to-volume ratio for cell infiltration and attachment, facilitating nutrient diffusion and vascularization while avoiding bacterial infections, an interconnected geometry and structural strength and be specific to the shape of the wound [17,36,37].

**Figure 1:**
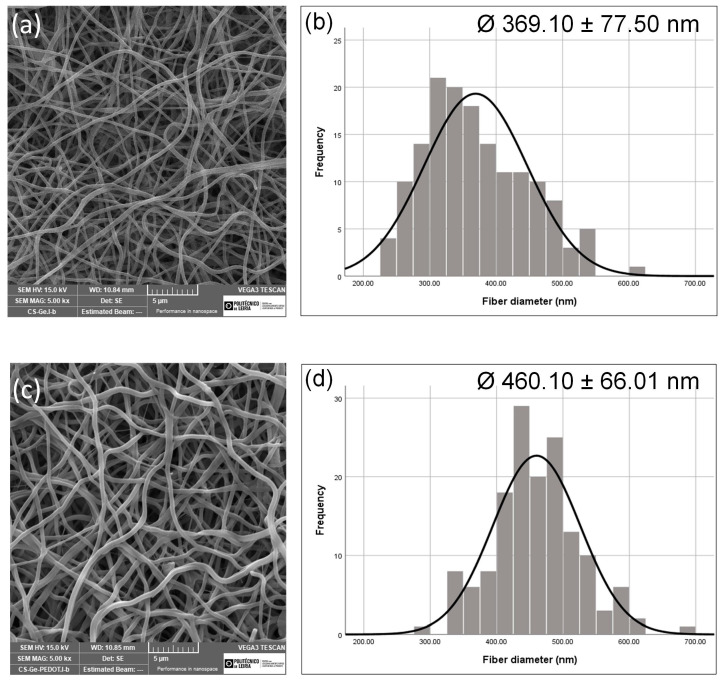
(**a**) Electrospun CS/GEL nanofibrous meshes. (**b**) CS/GEL nanofibers’ diameter histogram distribution. (**c**) Electrospun CS/GEL/PEDOT nanofibrous meshes. (**d**) CS/GEL/PEDOT nanofibers’ diameter histogram distribution. Scale bars correspond to 5 µm.

Morphological images of the electrospun CS/GEL and CS/GEL/PEDOT meshes taken using SEM are shown in Figure 1, along with their diameter distribution. The micrograph image depicts a randomly aligned, interconnected porous structure for both the CS/GEL and CS/GEL/PEDOT nanofibers. The range of the CS/GEL samples was estimated to be between 292–446 nm, with a mean value of 369.1 ± 77.5 nm, while the diameter of theCS/GEL/PEDOT nanofibers’ diameter was estimated to be between 394–526 nm, with a mean value of 460.1 ± 66.0 nm, without statistical significance for the presence of PEDOT (*p* > 0.05). The fact that different voltages were used to obtain the two sets of nanofibers greatly difficult the comparison between the diameter obtained for the CS/GEL/PEDOT and CS/GEL samples, and that limits the assumption that the incorporation of PEDOT results in a higher diameter. However, during electrospinning, a higher applied voltage leads to a greater stretching of the solution and that would normally translate into thinner fibers [38]. However, since the CS/GEL/PEDOT samples were obtained using a higher voltage and still possess a higher diameter than CS/GEL samples, this could mean that the addition of PEDOT compensated for the decrease in the diameter that results from the increase in voltage. These results may be related to the conductivity of the solutions, given that the CS/GEL/PEDOT solution has a conductivity of 2306.00 ± 11.40 µS and the CS/GEL solution of 253.80 ± 1.48 µS (approximately 10 times lower), which is the main reason for the difference in the voltage used for nanofiber production. The estimated apparent density value of the CS/GEL meshes was 0.23 ± 0.02 g/cm^3^ while the apparent density of the CS/GEL/PEDOT was 0.49 ± 0.05 g/cm^3^, which sustains the hypothesis that such a shift in density is due to the incorporation of PEDOT and that can, in fact, compensate for the increase in voltage during electrospinning, permitting a slight increase in the nanofiber diameter. The formation of either droplets, beads or fibers depends on the CS/GEL and CS/GEL/PEDOT spinning solutions’ surface tension, as lower surface tension leads to the occurrence of electrospinning at lower electric fields [39]. The dissolution of chitosan is generally easy in diluted acid aqueous solutions such as 1 or 2 *v*/*v*.%; however, these are not electrospinnable solutions, independently of the chitosan concentration. The increase in the acetic acid concentration results in a decrease in surface tensions, thus decreasing the voltage required for the occurrence of electrospinning. One way to, in future, produce CS/GEL and CS/GEL/PEDOT nanofibers at equal voltage would be to maintain the acetic acid solution concentration for the CS/GEL solution and increase the acetic acid concentration in the latter. Additionally, the presence of PSS, a salt, is known to increase charge density on the surface of the jet and that translates into stronger whipping instability of the jet. The presence of salts is also known to increase mass flow and, consequently, fiber thickness [39,40,41]. Qin et al. [42] also stated that increased solution conductivity translated into higher fiber diameter, which they related to the increase in the surface charge of the spinning jet, making the spinning more fluent, increasing the ejected solution quantity and thus increasing fiber.

Attending to similar PEDOT synthesis and nanofiber production protocols, Kiristi et al. [43] successfully produced electrospun nanofibers from a CS/poly (vinylalcohol) (PVA)/PEDOT solution. In this work, CS was dissolved in 0.5 M acetic acid at 2 wt.%, PVA was dissolved in water at 10 wt.% concentration and PEDOT was dispersed in N-methyl-2-pyrolidone (NMP) at 1 wt.%. The three components, blended at a 1:1:1 volume ratio (CS/PVA/PEDOT), with a flow rate of 0.01 mL/h, an applied voltage of 24 kV and a tip-to-collector distance of 15 cm, yielded nanofibers with an average diameter of 200 ± 50 nm. When the ratio was increased to 2:1:1, the fiber diameter also increased to 246 ± 30 nm. The calculated porosity of the CS/GEL and CS/GEL/PEDOT electrospun meshes produced (Table 1) evidenced that the addition of PEDOT decreased the porosity of the nanofibers from 89.89 ± 0.09% to 77.14 ± 2.49%. This decrease in porosity can result from an increase in fiber packing due to an increase in apparent density of the CS/PEDOT meshes (from 0.23 ± 0.02 to 0.49 ± 0.05 g/cm^3^), as a result of the introduction of a dense and highly conjugated conductive polymer within the nanofibers’ structure. Despite not reducing water content uptake due to the presence of hydrophilic components such as CS and GEL, the decrease in porosity can also be correlated to the hydrophobic nature of PEDOT [44] and can affect the scaffold biological performance. The increase in diameter verified from the addition of PEDOT to the nanofibers (from 369.10 ± 77.50 nm to 460.10 ± 66.01 nm), translated into a decrease in porosity, which should be consistent with the notion that web density remained the same [44,45]. However, in Figure 1, it is noticeable the increase in fiber diameter resulted in an increased pore size and pore number as the fiber area-to-total area ratio (web density) decreased. This may be due to the difference in the voltage used, which derives from the conductive nature of the solutions; despite equal deposition times, the CS/GEL/PEDOT solution required a higher voltage for the production of fibers and that can, in fact, influence fiber packing, increasing it.

Both the decrease in sample porosity and an increase in apparent density with the incorporation of PEDOT has been shown to be statistically insignificant (*p*-value > 0.05). The presence of PEDOT in the electrospinning solution is responsible for an increase in solution conductivity that is statistically significant. Contrarily to what is expected, this increase in conductivity translates into a slightly higher diameter, which may be due to a decrease in surface tension and net charge density carried by the jet during the electrospinning process [46]. The difference in pH between the CS/GEL and the CS/GEL/PEDOT solutions has proven to be statistically insignificant. The decrease in pH resulting from the addition of PEDOT (from 2.897 ± 0.007 to 2.844 ± 0.004) is due to oxidative polymerization conducted under acidic conditions to enhance reaction efficiency, combined with the incorporations of negatively charged HA into the polymer backbone during doping.

### 3.2. Physicochemical and Structural Characterization

**Figure 2:**
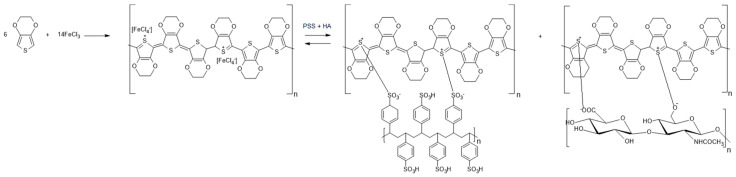
Schematic diagram of EDOT oxidation with FeCl_3_, originating PEDOT and tetrachloroferrate, following the addition of PSS and HA. PSS acts as a counterion for PEDOT neutralization during polymerization and hyaluronic acid acts as a p-type (oxidant) dopant, originating a charged PEDOT chain with delocalized electrons, which is thus conductive.

In order to evaluate the interactions between the intervenient components—chitosan, PEDOT, hyaluronic acid, gelatin and the crosslinker, BDDGE—Fourier transform infrared spectroscopy with attenuated total reflection (FTIR-ATR) analysis was performed on the CS/GEL and CS/GEL/PEDOT electrospun meshes. The CS/PEDOT composite produced through chemical oxidation of EDOT consists of a polycationic polymer, chitosan and PEDOT, obtained through in situ polymerization of EDOT with FeCl_3_ and doping with HA, but while the iron chloride was washed, the hyaluronic acid, which is a negatively charged molecule, is capable of binding itself to the polymer during the doping process [13]. In the same way, large molecules such as PSS (even if PSS only works as a counterion and not as a dopant) can become entrapped into the polymer and will not leach with time or with electrical stimulus application, granting the polymer greater electrochemical stability [13,25,47,48]. In this work, PSS is used as a counterion for PEDOT during polymerization, forming a stable dispersion in water and retaining its conductivity through the creation of a Polyelectrolyte Complex (PEC) [25], and HA is used as a dopant to provide biological activity and enhance conductivity [8]. Equal concentrations of both PSS and HA were used for a balanced distribution of polyanionic PSS for PEDOT stabilization and charged HA dopant [21,25,44] (Figure 2).

**Figure 3:**
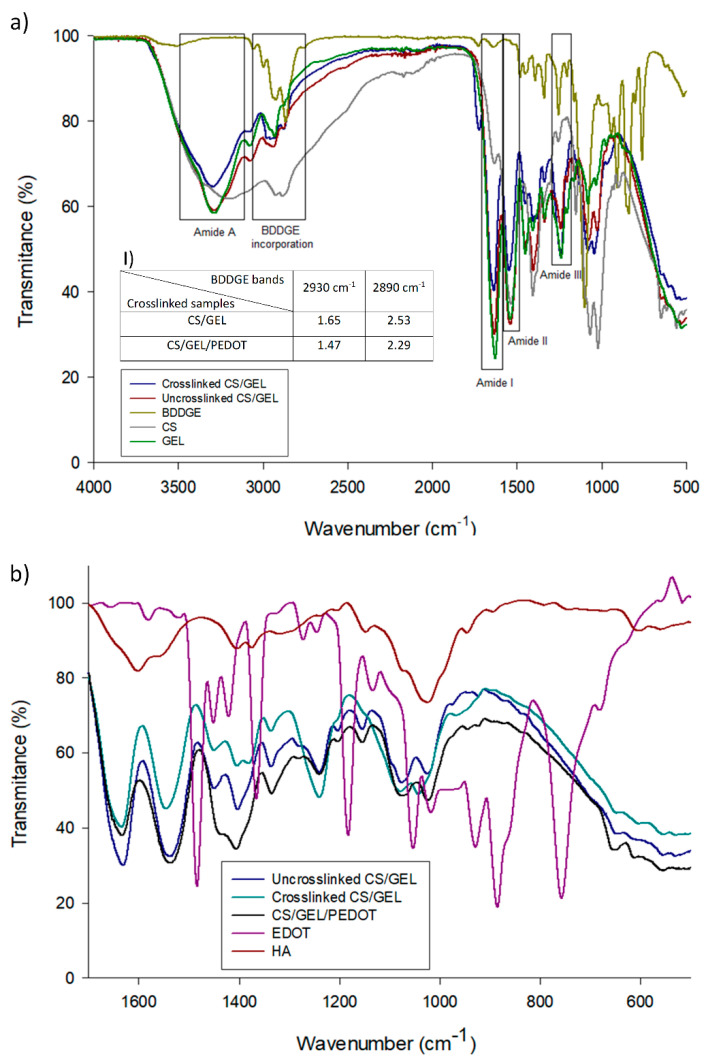
FTIR-ATR spectra of (**a**) uncrosslinked CS/GEL film, crosslinked CS/GEL and CS/GEL/PEDOT films, raw CS powder, raw GEL powder and raw BDDGE solution, (I) ratio between non-crosslinked and crosslinked samples of CS/GEL and CS/GEL/PEDOT at 2930 and 2890 cm^−1^, in which ratio ≥ 1 confirms the presence of BDDGE; (**b**) CS/GEL, HA-doped CS/GEL/PEDOT and undoped CS/GEL/PEDOT films, raw HA powder and raw EDOT solution, confirming the incorporation of HA during polymerization, as a charged dopant.

The different spectra obtained for both samples are shown in Figure 3a, suggesting no new covalent bonds originated and that the polymers interacted only by hydrogen and/or van der Waals forces, showing the signature spectra of the individual polymers in solution [49]. In the FTIR-ATR spectra of both the CS/GEL and CS/GEL/PEDOT samples, pure chitosan is characterized by the following bands: (1) a broad band between 3350–3200 cm^−1^ is attributed to the O–H and N–H stretching vibrations of chitosan, typically associated with intermolecular and intramolecular hydrogen bonding among hydroxyl and amino groups; (2) peaks at 2920 cm^−1^ and 2880 cm^−1^, owing to C-H stretching in asymmetric CH_2_ and symmetric CH_2_ or CH_3_ stretch, respectively; (3) a peak at 1629 cm^−1^, due to C=O stretching in the amide I; (4) a peak at 1534 cm^−1^, due to N-H bending in amide II; (5) a peak at 1412 cm^−1^ due C-N stretching vibration of amide III; and (6) the characteristic bands of the polysaccharide primary chain at 1150 cm^−1^, assigned to the asymmetrical stretch of the C-O-C bridge, 1066 cm^−1^, 1022 cm^−1^ and 894 cm^−1^, associated with skeletal vibrations involving C-O stretching [50,51,52,53]. In the samples containing gelatin, it is possible to observe four different amide regions: (1) a band in the range of 3120–3520 cm^−1^ assigned to amide A, which has a peak at 3280 cm^−1^, owing to the N-H stretching vibration and possibly to some OH stretching vibration due to presence of water; (2) a band between 1580–1700 cm^−1^ assigned to amide I, which has a peak at 1630 cm^−1^, owing to the carbonyl (C=O) stretching vibration and some minor C-N stretching vibration; (3) a band in the range of 1475–1590 cm^−1^, associated with amide II, peaking at 1545 cm^−1^; and (4) a band in the range of 1180–1300 cm^−1^, assigned to amide III, with a peak at 1245 cm^−1^, corresponding to amide III [1,50,54,55,56]. Amide II results from the contribution of the bending vibration of N-H groups and the stretching vibrations of C-N groups, determining the protein’s secondary structure. However, the amide III band can also contribute to the protein’s secondary structure as it presents in-plane vibrations from N-H bending, C-N stretching and the interaction between these two nodes, with weak contributions from C-C stretching and C-O in-plane bending, showing a less defined vibrational mode with varying protein vibration [1,57,58,59]. The greater similarity of the CS/GEL FTIR spectrum to raw gelatin’s spectra is visible; however, there is also a slight shift in the O-H and N-H bonds, at approximately 3000 cm^−1^, associated with chitosan. In the CS/GEL spectrum, a slight increase in the relative intensities of bands is also visible, corresponding to vibrations of the C-OH and C-O-C bonds (1200−1000 cm^−1^) of the primary chain, confirming chitosan crosslinking with BDDGE, mainly via hydroxyl groups at the C6 position of the glucosamine unit, according to the literature [60]. It is possible to observe the signature contribution of the aliphatic moieties from BDDGE, at 2930 and 2890 cm^−1^, with very weak switches of primary amines to secondary amines [61], confirming its incorporation into the CS/GEL matrix and the crosslinking of gelatin via carboxylic acid groups [1,29]. The decrease in peak intensity of the amides I and II at 1640 and 1540 cm^−1^ may be due to the possible formation of a chitosan–epoxy blend; the intensity of the characteristic peak at 3300–3500 cm^−1^ decreased and a strong stretching vibration at 1540 cm^−1^ was observed, which may be derived from the reaction of the primary amino group of chitosan with the epoxide group. The increase in intensity of the bands in the range of 1000–1200 cm^−1^ can also be related to C-O stretching and can also be caused by the appearance of secondary alcohol groups upon epoxy–amine reaction in gelatin [61]. The peak at 1070 cm^−1^ represented C–O stretching resulting from the cleavage of the oxirane ring by NH_2_. These data suggest that the samples are possibly crosslinked via carboxylic groups of gelatin, hydroxyl groups at the C6 position of the glucosamine unit of chitosan and terminal amino groups of chitosan through diepoxide linkage [1,53,60]; however, further studies on the quantification of crosslinking extent are necessary. Additionally, the presence of PEDOT blended with chitosan and gelatin is visible by three main peaks, associated with the bands of the thiophene ring, located between 1490 and 1360 cm^−1^ (aromatic C=C antisymmetric and symmetric stretching and quinoidal C-C stretching, respectively), and by two peaks, associated with the dioxane ring, at about 1050 cm^−1^ (C-O stretching) and 900 cm^−1^ (O-C-C deformation), suggesting the successful formation of PEDOT (Figure 3b) [25,44]. As evidenced in Figure 3b, the peak of HA at approximately 1630 cm^−1^, characteristic of its amide II group, overlaps with the bands of the amide I group in the CS/GEL and CS/GEL/PEDOT (derived from GEL), which suggests the incorporation of hyaluronic acid during the doping process [44]. In the FTIR-ATR spectrum of the CS/GEL/PEDOT composite synthesized without HA, the incorporation of PEDOT through the bands associated with the thiophene ring and a decrease in the intensity of the band associated with the amide carbonyl group that is characteristic of HA are visible. However, a great similarity is noticeable between the spectra of the CS/GEL/PEDOT films synthesized with and without HA due to the presence of CS and GEL, which promotes the overlapping of amides I, II and III bands from both polymers and makes difficult the analysis of each component’s contribution. Table 2 summarizes the identified bands and their assignments for each main polymer.

### 3.3. Water Uptake, Dissolvability and Water Vapor Permeability

Hydrophilicity is a very important factor in the design of scaffolds, as this characteristic determines their interaction with cells and surrounding tissues, influencing, amongst other aspects, their mechanical properties, their biodegradability in an aqueous environment through hydrolysis and their ability to maintain a moist environment in the wound bed for faster wound healing [1,62]. Table 1 shows the swelling degree in the presence or absence of PEDOT; uncrosslinked samples were evaluated as controls as well. In this work, the addition of BDDGE translated into a decrease in water uptake, during the first 24 h, from 6366.67 ± 556.43% to 1068.01 ± 55.72% and in a decrease in dissolvability from 52.12 ± 4.53 to 11.27 ± 1.16%, as the polymer chains come closer together due to new bond formation, increasing compaction through stronger retraction forces [29,30]. It was observed that the increase in the crosslinking time of the CS/GEL samples resulted in an increase in water uptake and dissolvability, evidencing that, with time, the stability of the chitosan and gelatin crosslinking decreases. This may be due to two side reactions: (1) increasingly more hydrophilic OH groups are generated as the outcome of the reaction of NH_2_ of chitosan with the oxirane ring of epoxy, counteracting the crosslinking reaction [53]; and (2) a decrease in pH over time leads to NH_2_ and COOH partial protonation (from chitosan and gelatin, respectively), limiting the bonding between BDDGE’s diepoxide groups and primary amino and carboxylic acid groups [1,60]. In fact, stabilization is influenced by a complex interplay of crosslinker concentration, incubation time and polymer accessibility.

The addition of PEDOT to the crosslinked CS/GEL meshes increased the electrospun meshes’ swelling degree and dissolvability. The presence of PSS in the synthesis solution and its entrapment into the CP’s chain, known to increase the solubility of the monomer and its stability in water during polymerization, is also responsible for the hydrophilicity of PEDOT, as polymers containing sulfonic acid groups tend to be very hygroscopic [25,63]. PEDOT is also doped with HA, a hygroscopic polymer responsible for modulating tissue hydration and osmotic balance, which can contribute to this behavior. The increase in crosslinking time in the CS/GEL/PEDOT samples also resulted in an increase in water uptake and in dissolvability, equally associated with a possible decrease in crosslinking due to side reactions of chitosan with BDDGE, as it crosslinks in both primary amino groups hydroxyl groups. The presence of PEDOT has shown to be statistically relevant in both dissolvability and water uptake, for 24 h, 48 h and 72 h of crosslinking, proving that incorporation of in situ polymerized PEDOT improves the meshes’ water absorption. For both conditions, the crosslinking reaction seems more stable at 24 h due to the lower dissolution and higher swelling degree.

Just as important as fluid absorption that enhances rehydration of necrotic tissues and promotes autolytic debridement of the wound, water loss through evaporation is a very important criterion for the design of wound dressings, especially for wounds with exudate, as it directly regulates the moisture of the microenvironment of wound healing [23]. Water loss from a granulating wound surface can increase as to twenty times as much than that of normal skin [64] and when the wound is directly exposed to air, it dehydrates and a scab is formed. Wound healing under moist conditions is known to be faster in comparison to dehydration conditions, however, an adequate scaffold must not retain excessive fluid nor allow wound bed dehydration, as exudate contains not only water but often cellular debris and enzymes, which can be harmful to the healthy tissue surrounding the wound and dehydration of traumatized or ischemic leads to further tissue loss by transforming the “zone of stasis” adjacent to the zone of injury into a “zone of necrosis” [65,66].

In this work, the water vapor permeability (WVP) value for the CS/GEL meshes is of 2715.09 ± 63.06 g/m^2^/day and for the CS/GEL/PEDOT meshes is of 2745.58 ± 15.27 g/m^2^/day, which are slightly above the typical optimal range (2000–2500 g/m^2^/day), however current evidence suggests that such a modest increase keep the adequate level of moisture, without risking wound dehydration [65,67]. The WVP of dressings is influenced by fiber diameter, pore size and pore interconnectivity; meshes with small pores and packed fibers exhibit lower WVP values [1]. From Table 1 it is possible to observe that WVP values increase with the addition of PEDOT which can be correlated with both the increase in the fiber diameter and the decrease in nanofiber compaction, but without statistical significance.

### 3.4. Hydrolytic and Enzymatic Degradation

**Figure 4:**
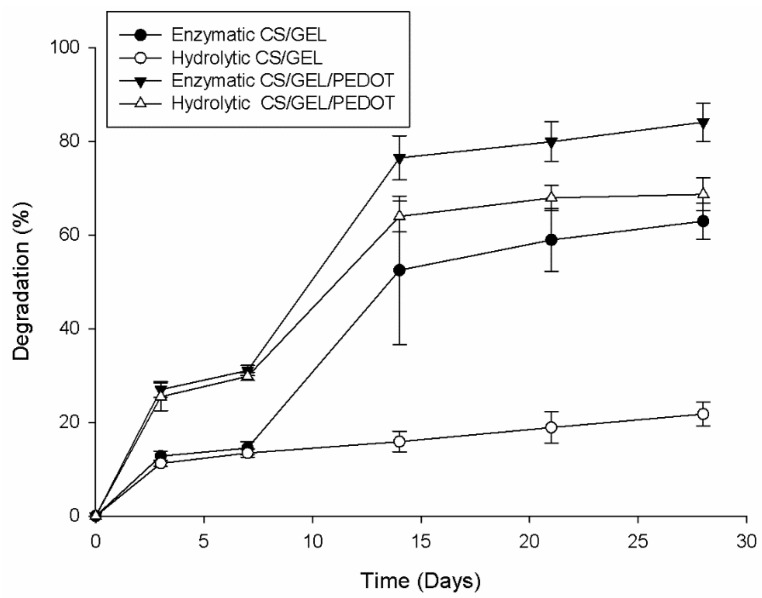
Degradation kinetics over 28 days.

A scaffold’s biodegradability rate is one of its most important characteristics, as it should degrade at the same rate as the tissue it aims to reconstruct regenerates [13]. Given this, control and conductive samples were evaluated regarding their hydrolytic and enzymatic degradation (Figure 4) in order to quantify the weight loss over 28 days, as a means to assess the capability of HA of increasing PEDOT’s biodegradability through the doping process.

The hydrolytic degradation of the CS/GEL samples evidences a steady increase and low degradability over time until a maximum of 21.78 ± 2.59% reached after 28 days, while the CS/GEL/PEDOT samples did not exhibit relevant hydrolytic degradation for the first two time-points (3 and 7 days), reaching only a value of 29.81 ± 0.87%. When comparing the values of control and conductive samples in the first time-point (3 days) of the hydrolytic degradation, we can observe that they are very similar to the values calculated for dissolvability during 24 h, for samples also crosslinked for 24 h: the dissolvability of control samples was 11.27 ± 1.16% and their hydrolytic degradation value at 3 days was 11.25 ± 0.66%, while for the conductive samples dissolvability was 26.48 ± 1.78% and hydrolytic degradation in the first time-point was 25.46 ± 2.94%. The similarity of these values sustains the hypothesis that the loss of mass is associated once more with an incomplete/reversible crosslinking and its possible side reactions rather than an effective degradation per se.

After 14 days, the conductive samples experience an accentuated degradation up to 63.96 ± 3.32%, reaching 68.69 ± 3.50% at the end of the 28 days. This sudden increase in hydrolytic degradation is probably due to the samples’ incomplete crosslinking, which allows the gradual exposure of the mesh to the medium, as time elapses and continuously potentiates its susceptibility to degradation. Similarly, in the enzymatic degradation of both the control and conductive samples, exponential degradation occurred only after 14 days. At time-point 2 (7 days), the degradation values for the CS/GEL meshes were 14.51 ± 1.37% and those for the CS/GEL/PEDOT were 31.07 ± 1.09% and they increased at time-point 3 (14 days) to 52.47 ± 5.38% and 76.48 ± 4.71%, respectively. This may be due to nanofiber distribution heterogeneity, as some parts of the mesh can be more compact than others, making difficult its permeation by the medium and altering the degradation profile. Despite the porosity being the highest in the CS/GEL samples, the CS/GEL/PEDOT composite present in the conductive samples was synthesized in the presence of chitosan and hyaluronic acid, increasing the resulting polymer’s biodegradability, as suggested in the literature [18].

Acid hydrolysis of the glycosidic linkages of chitosan can alter the solution’s pH, involving the following steps: (i) protonation of oxygen at the glycosidic linkage; (ii) addition of water to the reducing sugar end group; and (iii) decomposition of the protonated glycosidic linkages. The catalytic protons may be present in the water contained in the samples, and the protonated amino group of chitosan may probably also act as a proton donor in the catalysis. The amine groups initially became protonated by H^+^ and then the excess value of acid catalyzes the reaction. Variations in enzymatic degradation can also be related to media pH. Lysozyme’s activity is pH- and ionic strength-dependent within a range. The activity of lysozyme is a function of pH over a broad pH range (6.0–9.0), and at pH 6.2, maximal activity is observed over a wider range of ionic strengths (0.02–0.100 M) than at pH 9.2 (0.01–0.06 M). Enzymatic degradation of chitosan with a high deacetylation degree (95–98%) results in lower weight loss due to the low sensitivity of amine groups to lysozyme [34]. The superior value of gelatin in the solution translates into the stabilization of chitosan’s degradation, as gelatin tends to decrease the total weight loss of the blend [68].

### 3.5. Contact Angle

The contact angle values present in Table 1 and Figure S1 evidence the hydrophilicity of the electrospun meshes, as expected. The addition of PEDOT to the samples translated into an nonrepresentative increase in the contact angle at 0 s, delaying water absorption by the structure, in the first seconds, which may be related to an increase in hydrophobicity [44]. At 0 s, the CS/GEL samples evidenced an average contact angle of 20.06 ± 1.77°, which, after 5 s, decreased to 0°, just like the CS/GEL/PEDOT samples that started with an average contact angle of 21.76 ± 1.78°, which, after the same amount of time, decrease to 0°. Even if the presence of PEDOT/PSS does relate to higher hydrophilicity, the presence of PEDOT nanoparticles anisotropically dispersed in the nanofibrous mesh and the nanofiber assembly, which includes the effect of microscopic air pockets trapped below the liquid droplet, can lead to a hydrophobic interface, thus impeding the penetration of the water droplet into the highly porous structure [69].

### 3.6. Mechanical Properties

**Figure 5:**
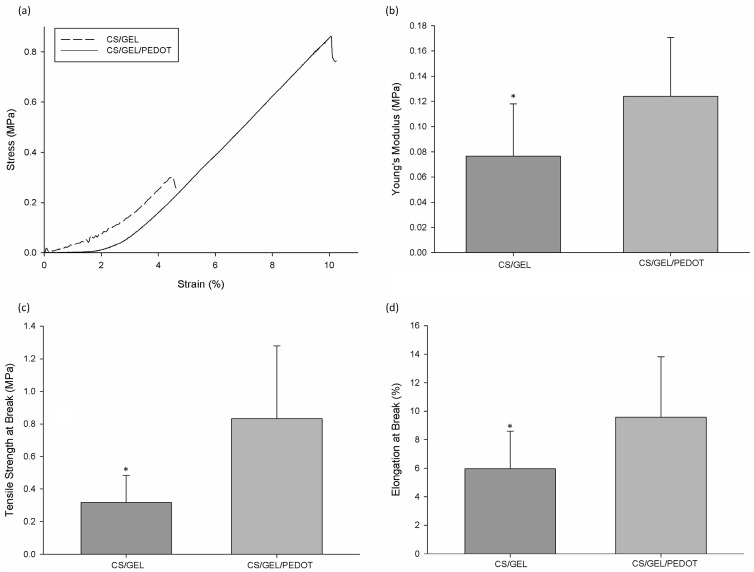
Mechanical behaviour of electrospun CS/GEL and CS/GEL/PEDOT crosslinked samples in wet state, (**a**) stress–strain representative curves; (**b**) Young’s modulus; (**c**) tensile strength at break; (**d**) elongation at break. Statistical significance for *p* ≥ 0.05 (*).

The mechanical properties of the electrospun CS/GEL meshes were also investigated as a function of the presence of PEDOT. Representative stress–strain curves for the samples tested in the wet state are shown in Figure 5a. From a total of 15 of each of those curves, it was possible to obtain the Young’s modulus, the tensile strength at break and the elongation at break. According to Dias et al. [1], electrospun GEL nanofibers (15 wt.%) crosslinked with 4 wt.% BDDGE for 24 h presented an average Young’s modulus of 0.67± 0.45 MPa, a tensile strength of 0.16 ± 0.15 MPa and an elongation at break of 45.01 ± 9.46%. CS/GEL electrospun meshes presented an average Young’s modulus of 0.08 ± 0.04 MPa, a tensile strength at break of 0.32 ± 0.17 MPa and an elongation at break of 5.97 ± 2.64%, which means that chitosan only benefited the tensile strength of the samples, making them more ductile. In parallel, the conductive samples presented an average Young’s modulus of 0.12 ± 0.05 MPa, a tensile strength at break of 0.83 ± 0.45 MPa and an elongation at break of 9.58 ± 4.25% for those same characteristics, respectively. CS/GEL/PEDOT exhibit higher values for the three properties, which suggests that the incorporation of the conductive polymer translates into a more robust sturdier, more flexible and less brittle electrospun meshes in comparison to CS/GEL samples. The increase in elongation at break is in accordance with a possible lower crosslinking degree, leading to a less dense and less compact structure [1]. Moreover, the increase in tensile strength at break and elongation at break can also be due to the plasticizing effect provided by the secondary hydroxyl groups and hydroxyl-terminated pendant groups from hydrolyzed un-reacted epoxides of BDDGE and hydroxyl groups of hyaluronic acid-doped PEDOT [1,29]. The increase in Young’s modulus is due to a strain stiffening behavior, originated by PEDOT crystallites; straining leads to an irreversible change in fiber orientation, accompanied by a pronounced increase in the elastic modulus of the samples [70,71]

As a reference for the human skin’s Young’s modulus, tensile strength and elongation at break, we considered 2.9–150 MPa, 1–32 MPa for tensile strength and 17–207%, respectively [1]. In terms of mechanical properties, the CS/GEL/PEDOT electrospun meshes exhibited lower values, when compared to human skin, accentuating the importance of developing hybrid structures to mimic the reference mechanical properties through a combination of other materials, for example, natural and synthetic polymer blends and other processing techniques [72,73].

## 4. Conclusions

This work is, to best of our knowledge, an unique approach to PEDOT in in situ polymerization, doping and conductive nanofiber production. We have selected a very specific set of reagents in order to establish a novel biodegradable PEDOT composite, combining natural polymers such as chitosan, gelatin and hyaluronic acid to enhance the biodegradability of PEDOT for tissue engineering applications. The main purpose of this research work was to explore the potential of in situ polymerization and electrospun electrostimulated wound dressings for skin regeneration. We were able to synthesize a biodegradable chitosan/PEDOT composite and incorporate it into an electrospinnable solution in order to produce conductive chitosan/gelatin fibers. The conductive electrospun meshes were characterized and tested against the control condition, evidencing particular properties from the combination of CS and GEL and their crosslinking reaction and the influence of PEDOT in physicochemical and mechanical properties of the nanofibers. The addition of chitosan to the gelatin solution creates a complex crosslinking system that requires further study to comprehend precisely how it takes place and to what extent. Overall, this study focused on the electrospinning and characterization of polymeric CS/GEL nanofibers incorporated with hyaluronic acid-doped PEDOT obtained through an alternative route for PEDOT.

## Figures and Tables

**Table 1:** Characterization of the electrospun CS/GEL and CS/GEL/PEDOT meshes. WVP—water vapor permeability, CA—contact angle, YM—Young’s modulus, TSB—tensile strength at break, EB—elongation at break.

Parameter		Sample	
		CS/GEL	CS/GEL/PEDOT
Porosity (%)		89.88 ± 0.88	77.14 ± 2.50
WVP (g/m^2^/day)		2715.09 ± 63.06	2745.58 ± 15.27
Solution pH		2.90 ± 0.01	2.84 ± 0.01
Solution conductivity (µS)		253.8 ± 1.48	2306 ± 11.4
Sample resistance (mΩ/Sq)		0	<100
Swelling degree (%)	24 h	1068.00 ± 55.72	1372.21 ± 73.75
	48 h	1075.20 ± 52.19	1377.11 ± 93.08
	72 h	1111.35 ± 50.60	1535.94 ± 100.99
Dissolvability (%)	24 h	11.27 ± 1.16	26.48 ± 2.08
	48 h	12.36 ± 1.25	30.48 ± 2.48
	72 h	13.23 ± 1.64	31.72 ± 2.74
CA (°)		20.06 ± 1.77	21.76 ± 1.78
YM (MPa)		0.08 ± 0.04	0.12 ± 0.05
TSB (MPa)		0.32 ± 0.17	0.83 ± 0.45
EB (%)		5.97 ± 2.64	9.58 ± 4.25

**Table 2:** Comparative FTIR bands and assignments to each main polymers used. CS—Chitosan, GEL—Gelatin and PEDOT—poly(3,4-ethylenedioxythiophene).

Polymers	Bands	Assignment
CS	3350–3200 cm^−1^	O–H and N–H stretching vibrations
	2920 cm^−1^	asymmetric CH_2_
	2880 cm^−1^	symmetric CH_2_ or CH_3_ stretch
	1629 cm^−1^	C=O stretching in the amide I
	1534 cm^−1^	N-H bending in amide II
	1412 cm^−1^	C-N stretching vibration of amide III
	1150 cm^−1^	asymmetrical stretch of the C-O-C bridge
	1066 cm^−1^, 1022 cm^−1^ and 894 cm^−1^	skeletal vibrations involving C-O stretching
GEL	3120–3520 cm^−1^	amide A
	1580–1700 cm^−1^	amide I
	1475–1590 cm^−1^	amide II
	1180–1300 cm^−1^	amide III
PEDOT	1490 and 1360 cm^−1^	thiophene ring
	1050 cm^−1^ and 900 cm^−1^	dioxane ring

## Data Availability

Data is contained within the article.

## Supplementary Materials

The following supporting information can be downloaded at: https://www.mdpi.com/article/10.3390/polym17162227/s1, Figure S1: Representative contact angle measurements, (a) CS/GEL sample, (b) CS/GEL/PEDOT sample.
