# Supplementary material for: Electrospun PEDOT-Based Meshes for Skin Regeneration
Source: Polymers (Basel). 2025 Aug 15;17(16):2227. doi: 10.3390/polym17162227 (PMC12389447; doi:10.3390/polym17162227)
Supplement: Supplementary file 1 [file polymers-17-02227-s001.zip › polymers-3658759-supplementary.pdf]

Supplementary data

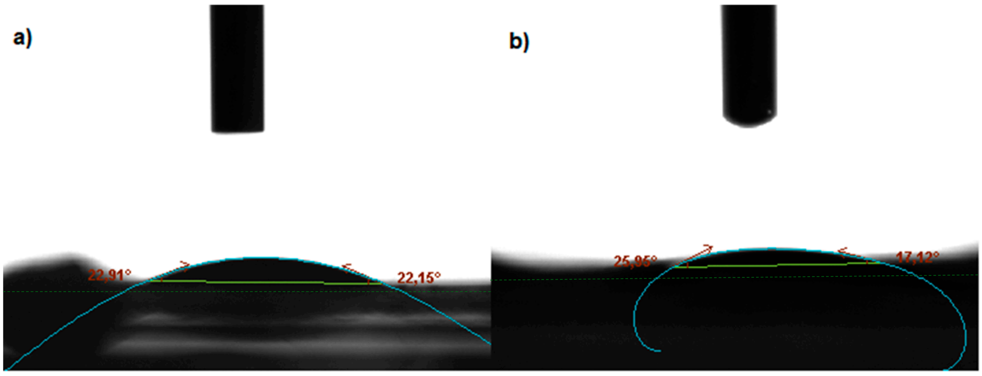

Figure S1 – Representative contact angle measurements, a) CS/GEL sample, b) CS/GEL/PEDOT sample.
